# History Taking in Non-Acute Vestibular Symptoms: A 4-Step Approach

**DOI:** 10.3390/jcm10245726

**Published:** 2021-12-07

**Authors:** Raymond van de Berg, Herman Kingma

**Affiliations:** Division of Balance Disorders, Department of Otorhinolaryngology and Head and Neck Surgery, Maastricht University Medical Center, 6229 HX Maastricht, The Netherlands; hermanuskingma@gmail.com

**Keywords:** medical history taking, dizziness, vertigo, vestibular diseases, Menière’s disease, benign paroxysmal positional vertigo, migraine disorders, vestibular neuronitis

## Abstract

History taking is crucial in the diagnostic process for vestibular disorders. To facilitate the process, systems such as TiTrATE, SO STONED, and DISCOHAT have been used to describe the different paradigms; together, they address the most important aspects of history taking, viz. time course, triggers, and accompanying symptoms. However, multiple (vestibular) disorders may co-occur in the same patient. This complicates history taking, since the time course, triggers, and accompanying symptoms can vary, depending on the disorder. History taking can, therefore, be improved by addressing the important aspects of each co-occurring vestibular disorder separately. The aim of this document is to describe a 4-step approach for improving history taking in patients with non-acute vestibular symptoms, by guiding the clinician and the patient through the history taking process. It involves a systematic approach that explicitly identifies all co-occurring vestibular disorders in the same patient, and which addresses each of these vestibular disorders separately. The four steps are: (1) describing any attack(s) of vertigo and/or dizziness; (2) describing any chronic vestibular symptoms; (3) screening for functional, psychological, and psychiatric co-morbidity; (4) establishing a comprehensive diagnosis, including all possible co-occurring (vestibular) disorders. In addition, pearls and pitfalls will be discussed separately for each step.

## 1. Introduction

History taking is a crucial aspect in the diagnostic process for vestibular disorders [1], given that symptomatology plays a key role in the diagnostic criteria [2,3,4,5,6,7,8,9,10,11,12,13]. If history taking is done properly, in combination with physical examination, a diagnosis can be made in many cases, even without additional laboratory tests or imaging. However, in many healthcare settings, history taking tends to be poor, and clinicians might be over-reliant on additional testing, resulting in misdiagnoses [14] and ineffective treatments. It is, therefore, imperative to improve the quality of history taking. To this end, various approaches have been proposed, e.g., asking key questions [15,16], focusing on time course and triggers (TiTrATE paradigm, [17]), on key dimensions (SO STONED paradigm, [18]), and on key symptoms (DISCOHAT paradigm, [19]).

The traditional, widespread approach in history taking is to focus on the quality of the symptoms, e.g., vertigo, presyncope, disequilibrium, or non-specific dizziness. Unfortunately, this approach has proved unreliable, for a variety of reasons: terms like ‘vertigo’ and ‘dizziness’ have different meanings to different people and in different languages; patients often have problems defining their symptoms; symptom descriptions might change over time; and the quality of the symptoms has little discriminative value [1,14,20,21]. This was why the TiTrATE (timing, triggers and targeted examination) paradigm was proposed, to emphasize the importance of the time course and triggers of symptoms in history taking. Focusing on the time course enables symptoms to be categorized into three major syndromes: acute vestibular syndrome, episodic vestibular syndrome, and chronic vestibular syndrome (Table 1) [17]. These syndromes can be subdivided into triggered or untriggered syndromes. For example, benign paroxysmal positional vertigo (BPPV) is an episodic vestibular syndrome triggered by head motion, while, Menière’s disease is an episodic vestibular syndrome without any trigger. TiTrATE also gives a quick overview of less urgent and more urgent differential diagnoses in one of the three syndromes (e.g., episodic vestibular syndrome, triggered: this might include BPPV (less urgent) or central positioning nystagmus due to a posterior fossa tumor (more urgent)) [17].

However, many vestibular disorders can present with a variety of accompanying otological and/or neurological symptoms, which can help in differentiating between disorders. For example, the presence of migrainous features during a vertigo attack might point to vestibular migraine [6], while the presence of fluctuating aural symptoms might point to Menière’s disease [7] and the presence of dysarthria or diplopia to a stroke [23]. Good history taking, therefore, goes beyond mere time course and triggers. The SO STONED paradigm was developed as a complementary tool, with a view to systematically acquiring eight key dimensions during history taking (since when, how often, symptom quality, triggers, otological symptoms, neurological symptoms, evolution, duration) [18]. In relation to the TiTrATE paradigm, the dimensions ‘how often’ and ‘triggers’ should be emphasized, since these are exactly the same as the ‘time course and triggers’ mentioned above; they too enable symptoms to be categorized into acute, episodic, or chronic vestibular syndromes. In addition, the DISCOHAT paradigm is specifically able to capture the wide spectrum of symptoms related to vestibulopathy (darkness worsens symptoms, imbalance, supermarket effect, cognitive complaints, oscillopsia, head movements worsen symptoms, autonomic complaints, tiredness) [19].

Combining the emphasis on time course and triggers (TiTrATE) with a systematic evaluation of all relevant dimensions (SO STONED) and specific symptoms (DISCOHAT) enhances the quality of history taking. Nevertheless, there remains an additional major challenge in almost half of all patients with vestibular disorders: the existence of co-occurring disorders; either primary or secondary [24,25]. The fact is that acute, episodic, and chronic vestibular disorders can co-occur, as can two or more disorders within the same syndrome type. Furthermore, functional, psychological, and psychiatric co-morbidity can, depending on the setting, be found in almost half of all patients as a primary diagnosis; or as a secondary diagnosis, which is often triggered by an acute or episodic vestibular event [8,25,26]. These factors complicate the use of the TiTrATE, SO STONED, and DISCOHAT paradigms, since co-occurring vestibular disorders might result in multiple different answers being given to the same question during history taking. A simple question about triggers might elicit answers (from the same patient) that would point to different disorders: e.g., ‘suddenly’ and ‘when I move my head very fast’ and ‘when I visit busy places’. As mentioned above, patients often struggle to describe (and to differentiate between) the different types of dizziness, and spinning and non-spinning vertigo [14], and tend to regard all their symptoms as stemming from ‘the same problem’. The clinician has to categorize all these symptoms into one or more vestibular syndrome and disorder.

The aim of this article is to propose a systematic approach to history taking in patients presenting with non-acute vestibular symptoms, consisting of four steps. It sets out to identify all vestibular disorders occurring at the same time in the same patient (e.g., BPPV and co-occurring persistent postural perceptual dizziness), by guiding the clinician and the patient through the process of history taking. This is important, since detecting all the disorders present in a single patient might have therapeutic implications, because it requires a multi-modal response (e.g., BPPV is treated differently from persistent postural perceptual dizziness) [27].

## 2. Overview of the 4-Step Approach

**Figure 1:**
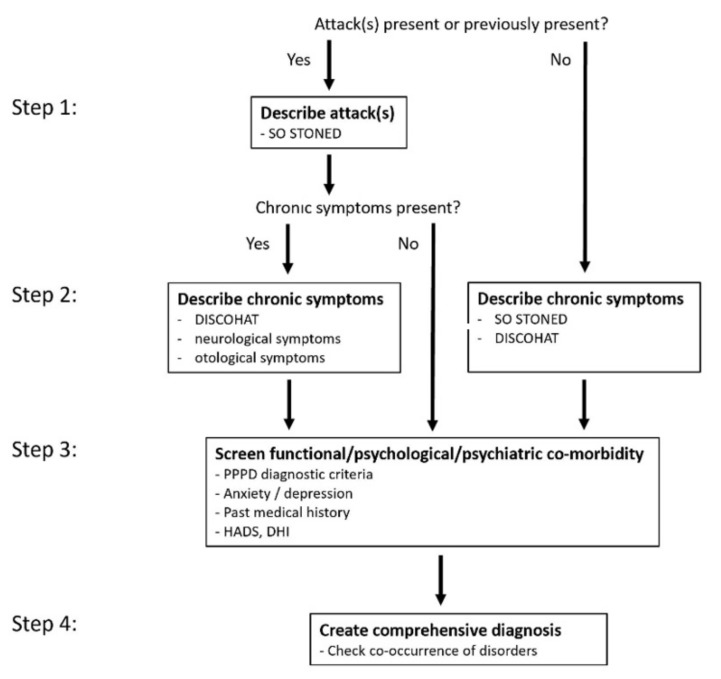
The 4-step approach to history taking in patients with non-acute vestibular symptoms. Each step investigates different aspects of vestibular disorders, while focusing on ‘one aspect at a time’. It explicitly screens for acute and episodic vestibular syndromes (step 1); chronic vestibular syndromes (step 2); and functional, psychological, and psychiatric co-morbidities (step 3). The aim is to identify all vestibular disorders occurring at the same time in the same patient, in order to create a comprehensive diagnosis (step 4). The ‘O’ and ‘T’ of ‘SO STONED’ are underlined, to emphasize the importance of paying specific attention to the aspects ‘how Often’ (=time course) and ‘Triggers’ of symptoms. SO STONED = acronym of ‘Since when, how Often, Symptom quality, Triggers, Otological symptoms, Neurological symptoms, Evolution, Duration’; DISCOHAT = acronym of ‘Darkness worsens symptoms, Imbalance, Supermarket effect, Cognitive complaints, Oscillopsia, Head movements worsen symptoms, Autonomic complaints, Tiredness’; PPPD = Persistent Postural Perceptual Dizziness; HADS = Hospital Anxiety and Depression Scale; DHI = Dizziness Handicap Inventory.

Figure 1 illustrates the 4-step approach to history taking.

- The first step is to investigate whether there are, or have been, any attacks of vertigo and/or dizziness. If so, the attacks are described.
- The second step involves exploring any chronic vestibular symptoms.
- The third step screens for functional, psychological, and/or psychiatric co-morbidity.
- During the fourth step, the diagnosis is established by explicitly taking into account the possibility of multiple co-occurring vestibular disorders.

### Key Points of the 4-Step Approach

The clinician and the patient are guided through the four steps, in order to systematically assess the (vestibular) symptoms.

Each step investigates different aspects of vestibular disorders: (1) attacks of vertigo and/or dizziness (=acute and/or episodic vestibular syndromes); (2) chronic vestibular symptoms (=chronic vestibular syndromes); (3) functional/psychological/psychiatric co-morbidity; (4) co-occurrence of various vestibular disorders. This systematic approach increases the chance of detecting the presence of multiple disorders, each with their own symptom profile (especially when practiced by less experienced clinicians, such as residents).

History taking focuses on ‘one aspect at a time’: either attacks, or chronic symptoms, or functional/psychological/psychiatric symptoms. This ‘forces’ the patient to discuss the relevant dimensions of that aspect alone, leading to a more accurate symptom description of that specific aspect. For example, when explicitly discussing vestibular migraine attacks, a patient is less likely to mention the unrelated tinnitus that is present only when lying in bed.

In the 4-step-approach, attacks of vertigo and/or dizziness are described first, even if they have already ceased. This is because describing the attacks might make it easier to understand the etiology. For example, a patient may initially report only the symptoms of vestibular hypofunction, but they may in fact be sequelae of recurrent attacks of vertigo that occurred previously, leading to bilateral vestibulopathy [28,29].

Steps two and three acknowledge that, for instance, the symptoms of uni- and bilateral vestibular hypofunction can be disabling [30,31,32] and that many patients with vestibular symptoms might suffer from functional/psychological/psychiatric co-morbidity [26].

## 3. Background, Pearls, and Pitfalls of the 4-Step Approach

### 3.1. Step 1: Describe Any Attack(s) of Vertigo and/or Dizziness

#### 3.1.1. Background

First, investigate whether one or more vertigo and/or dizziness attacks are present or have occurred in the past. If so, describe them using the SO STONED paradigm, with the emphasis on how often symptoms appear (=time course) and what triggers them. As described above, the SO STONED dimensions are: since when, how often, symptom quality, triggers, otological symptoms, neurological symptoms, evolution, duration. Please remember: the quality of symptoms (vertigo, dizziness, disequilibrium, etc.) is too unspecific to serve as a basis for a reliable diagnosis [1,14]; e.g., the absence of vertigo does not rule out a peripheral vestibular disorder [33].

The main otological symptoms are hearing loss, tinnitus, aural pressure, sound or pressure induced vertigo, hyperacusis, and symptoms of bone-conduction hyperacusis (e.g., autophony; hearing one’s eye movements) [13]. For neurological symptoms, special attention should be paid to migraine features and the ‘deadly Ds’ (dysarthria, diplopia, dysphagia, dysphonia, dysmetria, dysesthesia). In the acute setting (beyond the scope of this article), be aware of sudden, severe, or sustained pain, especially in the posterior neck region [6,23].

#### 3.1.2. Pearls and Pitfalls

Make sure the patient knows that the first questions will refer to the attack(s), and not to symptoms experienced during other circumstances. The patient must understand that the aim is to describe the attack(s) only.

It may be helpful to introduce each question with the words ‘During an attack….’. Some patients tend to forget that the questions during this first step refer only to the attack(s). 

Where there are multiple attacks with (almost) the same features, try to discuss an ‘average attack’. This can save time and avoids a comprehensive description of each and every attack. It also gives the patient the opportunity to mention that some symptoms are not always present (e.g., migraine features do not have to be present with each attack) [6] and that attacks might be of different durations, e.g., minimum 5 min to maximum 5 h.

Use a broad range of questions to investigate the triggers, since not all patients will know what is meant by triggers. If the patient cannot determine a trigger, other questions might help, e.g., avoidance of certain situations. Please note: there is a difference between a trigger and something that worsens an existing symptom. For example, acute unilateral vestibulopathy/vestibular neuritis is untriggered, but head movements can worsen the existing symptoms.

A trigger is defined as a factor that directly initiates the attacks. Patients with, say, vestibular migraine or Menière’s disease might mention ‘periods of stress’ or ‘change of weather conditions’ as triggers. This is not fully correct. Although some of these factors are associated with attacks, they do not seem to directly initiate the attacks and should not be regarded as triggers [34].

Depending on time course and triggers, different types of attacks can be present in the same patient (e.g., BPPV after an acute unilateral vestibulopathy/vestibular neuritis). In these cases, the SO STONED paradigm is applied separately to each type of attack.

The absence of reported accompanying symptoms, such as hearing loss, tinnitus, or aural pressure, does not rule out Menière’s disease; during an attack, patients might feel too sick to pay attention to symptoms other than vertigo, nausea, and vomiting.

The presence of accompanying otological and neurological symptoms during an attack does not necessarily mean that these symptoms were initiated or modulated by the attack. Please clarify the relationship between these symptoms (which may be pre-existing) and the attack(s). For example, a BPPV patient might suffer from chronic tinnitus that is not related to BPPV attacks, while a patient with Menière’s disease can have chronic tinnitus that might change in relation to an attack of Menière’s disease.

A history of migraine can reliably be ascertained by systematically covering all the migraine criteria [6]; not simply by asking ‘Do you have migraine?’ After all, patients who report having migraine might not actually have migraine, and patients who deny a history of migraine might actually have it.

The duration of symptoms should be the duration of the ‘most severe symptoms’. Some patients tend to describe the full time period until they feel (almost) recovered. However, this does not always equate to the duration of each attack. For example, a one-week episode of vertigo might refer to: one week of multiple short attacks, such as BPPV; a vertigo attack lasting several hours with residual symptoms of disequilibrium for one week, such as Menière’s disease or vestibular migraine; a vertigo attack lasting one week, such as acute unilateral vestibulopathy/vestibular neuritis. 

Presenting different possible symptom scenarios could help patients who have difficulty describing their symptoms. For example, patients with BPPV might overestimate the duration of their vertigo, due to anxiety. In such cases, it can help to present the following two scenarios: ‘After rolling over in bed, does the severe spinning dizziness last for literally a couple of minutes?’, and ‘After rolling over in bed, does the severe spinning dizziness last for less than a minute and then you still feel dizzy for a couple of minutes?’. 

### 3.2. Step 2: Describe Any Chronic Vestibular Symptoms

#### 3.2.1. Background

The second step covers patients with and without attacks of vertigo and/or dizziness. 

For patients with attacks, these chronic symptoms are not related to the attacks themselves, but occur in between attacks. Therefore, tell the patient that this part of history taking focuses on all the symptoms that occur in between attacks. The DISCOHAT acronym can be used to evaluate chronic vestibular symptoms, especially those related to uni- or bilateral vestibulopathy. As described above, these symptoms are: darkness worsens symptoms, imbalance, supermarket effect (=visually induced dizziness: sensitivity to moving visual stimuli or complex patterns, which is part of PPPD (see below) [8]), cognitive complaints, oscillopsia, head movements worsen symptoms, autonomic complaints, and tiredness [19]. Additionally, screen for otological and neurological symptoms unrelated to the attacks. 

For patients without any attacks of vertigo and/or dizziness, the SO STONED paradigm and the DISCOHAT acronyms can be used in tandem to describe the chronic symptoms. In this group of patients, the time course and triggers are important (e.g., unsteadiness when walking or standing, such as cerebellar ataxia or bilateral vestibulopathy) [9], as are the otological and neurological symptoms (including the ‘deadly Ds’).

#### 3.2.2. Pearls and Pitfalls

History taking can be challenging, in that certain symptoms are difficult to identify with just one or two questions (e.g., visual auras, visually induced dizziness, oscillopsia). Where in doubt as to whether the patient has understood the concept correctly, consider asking the patient to describe the symptom in a specific situation. For visual auras, for example, the patient might describe eye floaters rather than a visual aura.

### 3.3. Step 3: Screen for Functional, Psychological, and Psychiatric Co-Morbidity

#### 3.3.1. Background

In this third step, history taking is crucial, since no additional testing is available, which is pathognomonic of these disorders [8]. During this third step, pay special attention to functional dizziness, for instance persistent postural perceptual dizziness (PPPD) and to signs of anxiety and depression, or a medical history of functional, psychological, and psychiatric disorders.

PPPD is an example of a functional disorder, which means it arises from a change in the mode of action of the brain; this is different from a psychiatric disorder. Symptoms include persistent dizziness, unsteadiness, and non-spinning vertigo exacerbated by body movements or perceived movements, such as visually complex moving stimuli (=visually induced dizziness) [8].

Diagnostic keys of functional, psychological, and psychiatric co-morbidity include the following: symptoms congruent with the diagnostic criteria of PPPD [8]; chronic vestibular symptoms lasting for months or longer; fear of falls without falling; decrease of symptoms during mental distraction or alcohol intake; dizziness combined with disabling symptoms of hypersensitivity (e.g., disabling tinnitus and/or hyperacusis); disproportionate anxiety; situational triggers and avoidance behavior; symptoms of depression; chronic unsteadiness and dizziness after riding in a vehicle; a medical history of functional, psychological, and psychiatric disorders (e.g., anxiety disorder, depression, or somatic symptom disorders such as fibromyalgia) [35]. History taking in combination with physical examination and/or laboratory testing may reveal a dissociation between objective test results and the severity of subjective symptoms. Although abnormalities in vestibular testing do not correlate well with reported symptoms [36,37], it is possible to build a general ‘frame of reference’ to understand which symptoms (and their associated severity) might match which vestibular testing outcomes. For example, an objectively measured unilateral vestibular hypofunction occurring six months after an acute unilateral vestibulopathy/vestibular neuritis might generate some DISCOHAT symptoms, but it would not prevent a patient returning to work, unless the job is physically highly demanding (e.g., professional sport) or there is a statutory ban (e.g., an airline pilot). This discrepancy between objective findings and subjective symptoms is often explained by a functional, psychological, and/or psychiatric co-morbidity.

#### 3.3.2. Pearls and Pitfalls

Additionally, question patients about their anxiety and balance during physical examination and laboratory testing. They may report fear of falling, imbalance, or general anxiety when undergoing tests, something that will not be reflected in objective test results.

Questionnaires such as the hospital anxiety and depression scale and the dizziness handicap inventory can provide useful insights into the possibility of anxiety and depression as co-morbidities, on the one hand, and the impact of dizziness on daily life, on the other [38,39]. It can be useful to run the questionnaire process prior to consultation, to give the clinician advance knowledge of these aspects and to facilitate history taking.

### 3.4. Step 4: Create a Comprehensive Diagnosis

#### 3.4.1. Background

**Figure 2:**
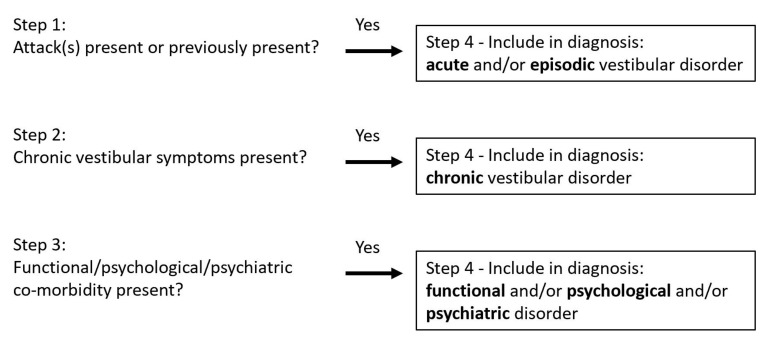
Using the 4-step approach to make a comprehensive diagnosis. Steps 1 to 3 investigate whether specific disorders might be added to the diagnosis. Finally, all co-occurring disorders are included in the diagnosis (step 4). Note: since the 4-step approach is aimed at patients presenting with non-acute vestibular symptoms, the ‘acute vestibular disorder’ mainly refers to a previous acute event (e.g., an acute unilateral vestibulopathy/vestibular neuritis that occurred five months previously).

In this final step, the diagnosis is established, explicitly taking into account the possibility of multiple co-occurring vestibular disorders. It includes evaluating all three previous steps, i.e., the presence of vertigo and/or dizziness attacks, chronic vestibular symptoms, and functional/psychological/psychiatric symptoms. Each step of this process evaluates whether an additional disorder should be added to the diagnosis. Figure 2 illustrates how a comprehensive diagnosis is arrived at by categorizing symptoms arising from all three previous steps into acute, episodic, and/or chronic vestibular disorders, and/or functional/psychological/psychiatric co-morbidity.

Significantly, this final step may result in three categories of disorders, all of which can occur at the same time in the same patient: (1) acute and/or episodic vestibular disorder(s); (2) chronic vestibular disorder(s); (3) functional/psychological/psychiatric co-morbidity. Since these disorders occur together, each category has its own list of differential diagnoses, with varying levels of urgency. For example, the differential diagnoses of episodic vestibular disorders include Menière’s disease, vestibular migraine, TIA, and arrhythmia, while the differential diagnoses of chronic vestibular disorders include vestibular hypofunction and cerebellar dizziness. PPPD is a chronic functional vestibular disorder which overlaps between chronic vestibular syndrome(s) and functional co-morbidity. 

Here is a practical example of the 4-step approach:

Step 1:
  For two years, symptoms of vertigo and dizziness, including multiple spontaneous attacks of vertigo.
  The average attack features:
    - Sudden sensation of vertigo
    - Triggers: none
    - Hearing loss on the left, aural pressure, and tinnitus (high-pitched sound)
    - No headache, no photo- or phonophobia, no visual auras, no migraines
    - No other neurological symptoms (including the ‘deadly Ds’)
    - Duration: minimum 5 min, maximum 2 h
    - Frequency: 2–5 times each month, frequency increased the last couple of months
Step 2:
  Chronic symptoms between attacks:
    - Darkness does not worsen symptoms; imbalance; supermarket effect (=visually induced dizziness); no problems with concentration or memory; no oscillopsia; fast head movements worsen symptoms; no autonomic complaints, not very tired
    - Persistent hearing loss and tinnitus on the left ear, no neurological symptoms
Step 3:
  - Significant distress resulting from chronic ‘fuzziness’ and the supermarket effect (=visually induced dizziness); no avoidance behavior or symptoms of anxiety or depression; no previous history of functional/psychological/psychiatric co-morbidity
Step 4:
  - Possible diagnosis based on history taking only: Menière’s disease on the left ear + vestibular hypofunction + PPPD

The four steps are explicitly represented in the example above:

1. Description of the attacks: symptoms might indicate Menière’s disease on the left ear.
2. Description of chronic symptoms: imbalance and stronger symptoms in relation to fast head movements might indicate vestibular hypofunction; visually induced dizziness might indicate PPPD.
3. Screening for functional, psychological, and psychiatric co-morbidity: apart from significant distress resulting from chronic ‘fuzziness’ and visually induced dizziness, which indicate PPPD, no indications of other functional/psychological/psychiatric co-morbidity.
4. Creating a comprehensive diagnosis: Menière’s disease, vestibular hypofunction, and PPPD all feature in the final diagnosis.

#### 3.4.2. Pearls and Pitfalls

Though the 4-step approach is useful in guiding the clinician towards a comprehensive diagnosis, this can only be a provisional diagnosis. Each disorder has its own variably urgent differential diagnoses. Physical examination and additional testing (laboratory tests, imaging, etc.) might be necessary to arrive at the final diagnosis.

The Bárány Society’s Committee for the Classification of Vestibular Disorders has defined consensus-based diagnostic criteria for most of the vestibular disorders [2,3,4,5,6,7,8,9,10,11,12,13]. These can be used to classify the reported symptomatology and to arrive at the proper diagnosis.

### 3.5. Other Relevant Questions

The 4-step approach focuses on vestibular disorders. However, other general aspects of history taking retain their importance. These aspects include use of medication and intoxicants (e.g., gentamicin, alcohol); other medical conditions and surgical procedures (e.g., auto-immune disorders, neurological disorders, ear surgery); family history (e.g., migraine, Menière’s disease [40]). Menière’s disease is often over-diagnosed [41]. A positive family history of Menière’s disease might not directly implicate Menière’s disease in the current patient; therefore, consider other (episodic) vestibular disorders, such as vestibular migraine.

## 4. First Experiences and Final Remarks

The 4-step approach to making a comprehensive diagnosis by guiding the clinician and patient through the process of history taking might at first seem like an unreasonably time-consuming process. The fact is, however, that history taking is a crucial aspect of the diagnostic process [1]. Investing time here might save time in the end, since it helps to establish the right (differential) diagnosis from the beginning of the process. History taking is a skill that can be trained and refined, much like surgery. These four steps require extensive training, but they will reduce the time needed to reach a diagnosis and increase efficiency. On completion of the requisite training, history taking with a ‘challenging’ patient in a tertiary referral clinic takes around 10–15 min. In secondary care centers, the time needed can be less for the majority of patients. The 4-step approach is, because of the time factor, less suited to patients presenting with acute vestibular symptoms.

The 4-step approach has been in use at a tertiary referral center in the Netherlands (Maastricht UMC+) for more than five years and has become part of the diagnostic process in other teaching hospitals. The initial experience is that more co-occurring disorders are being detected and the training of clinicians (e.g., residents) is improving under the 4-step systematic guidance. Structured discussion of (difficult) cases is on the increase, with the four steps featuring as smart phrases in hospital digital information systems.

A prospective randomized-controlled study might further validate the 4-step approach. This could, for example, comprise three comparable groups of residents (same specialty, same years of experience, etc.). Each group might then use a different paradigm for history taking in patients with vestibular symptoms: (1) the 4-step approach; (2) only SO STONED, with an emphasis on time course and triggers; (3) focusing on the quality of the symptoms. The diagnoses made by the residents could then be verified by experts in vestibular medicine. The main outcome measures might involve the correctness of the diagnoses made and the number of co-occurrent disorders detected.

Finally, the 4-step approach can be used globally to improve history taking in many vestibular patients, and it might also make people more aware of the importance of history taking and of the co-occurrence of vestibular disorders.

## Figures and Tables

**Table 1:** Vestibular syndromes categorized by time course and triggers. Modified and updated from [22].

Time Course	Trigger	Diagnosis: Less Urgent	Diagnosis: More Urgent
Acute vestibular syndrome	Spontaneous	Acute unilateral vestibulopathy/ vestibular neuritis Labyrinthitis	Stroke or hemorrhage Brainstem encephalitis Multiple sclerosis * Other internal/neuro
	Postexposure	Labyrinthine concussion	Skull base fracture Postoperative * Vertebral dissection Drugs (e.g., alcohol, anticonvulsants) Carbon monoxide intoxication Wernicke’s
Episodic vestibular syndrome	Spontaneous	Menière’s Disease Vestibular Migraine Vestibular Paroxysmia Vasovagal Panic *	Cardiac arrhythmia TIA (posterior circulation) Hypoglycemia *
	Trigger	Benign Paroxysmal Positional Vertigo Orthostatic hypotension Third mobile window syndromes * Superior canal dehiscence syndrome	Central positional nystagmus Vertebral artery compression/ occlusion syndrome
Chronic vestibular syndrome	Triggered or spontaneous	e.g., Vestibular hypofunction, Cerebellar dizziness, Functional dizziness	

*: can be less urgent or more urgent, depending on the case.
